# The Monetary Incentive Delay (MID) Task Induces Changes in Sensory Processing: ERP Evidence

**DOI:** 10.3389/fnhum.2019.00382

**Published:** 2019-11-01

**Authors:** Elena Krugliakova, Alexey Gorin, Tommaso Fedele, Yury Shtyrov, Victoria Moiseeva, Vasily Klucharev, Anna Shestakova

**Affiliations:** ^1^Centre for Cognition and Decision Making, Institute for Cognitive Neuroscience, National Research University Higher School of Economics, Moscow, Russia; ^2^Neurosurgery Department, University Hospital Zurich, Zurich, Switzerland; ^3^Department of Clinical Medicine, Center of Functionally Integrative Neuroscience (CFIN), Aarhus University, Aarhus, Denmark; ^4^Laboratory of Behavioural Neurodynamics, Saint Petersburg State University, Saint Petersburg, Russia

**Keywords:** neuroplasticity, attention, reinforcement learning (RL), feedback-related negativity (FRN), monetary incentive delay task, oddball paradigm, mismatch negativity (MMN), P3a

## Abstract

Numerous cognitive studies have demonstrated experience-induced plasticity in the primary sensory cortex, indicating that repeated decisions could modulate sensory processing. In this context, we investigated whether an auditory version of the monetary incentive delay (MID) task could change the neural processing of the incentive cues that code expected monetary outcomes. To study sensory plasticity, we presented the incentive cues as deviants during oddball sessions recorded before and after training in the two MID task sessions. We found that after 2 days of training in the MID task, incentive cues evoked a larger P3a (compared with the baseline condition), indicating there was an enhancement of the involuntary attention to the stimuli that predict rewards. At the individual level, the training-induced change of mismatch-related negativity was correlated with the amplitude of the feedback-related negativity (FRN) recorded during the first MID task session. Our results show that the MID task evokes plasticity changes in the auditory system associated with better passive discrimination of incentive cues and with enhanced involuntary attention switching towards these cues. Thus, the sensory processing of incentive cues is dynamically modulated by previous outcomes.

## Introduction

The traditional decision-making theory assumes that individuals’ choices are driven by values that are associated with prospective outcomes. Numerous neurobiological studies have implicated the involvement of dopaminergic neurons in the valuation stage of the decision-making process (Schultz, 2006) and in behavioral adaptations (Bromberg-Martin et al., 2010). Interestingly, popular neurobiological models of decision making (Rangel et al., 2008; Wang, 2012) acknowledge the key role of learning in reward-based decisions, but they indirectly assume that the primary sensory inputs to dopaminergic (decision making) networks are stationary and independent from previous decisions. However, many cognitive studies have demonstrated experience-induced plasticity in the primary sensory cortices (Atienza et al., 2005; Kujala and Näätänen, 2010; Shtyrov et al., 2010; Pantev and Herholz, 2011), indicating that repeated decisions could modulate sensory processing, which, in turn, could modulate follow-up decisions. In the current study, we tested the hypothesis that the repeated associations of a stimulus with a monetary outcome may evoke plasticity in an individual’s sensory processing. Furthermore, we tested the link between the neural activity underlying value-based learning and plastic changes in the sensory cortices.

Sensory cortices retain the capacity for experience-dependent changes, or plasticity, throughout life. These changes constitute the mechanism of perceptual learning (Gilbert et al., 2001). Numerous event-related potential (ERP) studies have shown training-induced neuroplastic changes in auditory information processing that could be explained by the reorganization of neuronal networks and changes in the sensitivity to and processing of relevant information (Atienza et al., 2005; Kujala and Näätänen, 2010; Shtyrov et al., 2010; Pantev and Herholz, 2011). In conditioning paradigms, where auditory tones are used as conditioning stimuli, the training results in associative representational plasticity, which selectively facilitates responses to the conditioned stimuli (Weinberger, 2007). According to the representational plasticity theory, the tuning of the neurons in the primary auditory cortex is selectively shifted towards the characteristics of the conditioned stimulus, thus biasing the whole sensory system to emphasize the behaviorally important stimulus (Diamond and Weinberger, 1986; Bakin and Weinberger, 1990; Edeline and Weinberger, 1993; for a review, see Weinberger, 2015).

The plasticity of auditory processing is often reflected in the mismatch negativity (MMN) component of auditory ERPs. The MMN is an electrophysiological signature of a pre-attentive process that detects alterations in a regular sound sequence (Näätänen, 1990; Winkler et al., 1996). The MMN is evoked by a deviant or rare (i.e., oddball) event embedded in a stream of repeated or familiar events (i.e., standards; Näätänen et al., 2007). The MMN is frequently explained in terms of predictive coding, which is a general theory of perceptual inference (Garrido et al., 2009; Carbajal and Malmierca, 2018). According to this theory, the brain actively learns the regularities of the sensory input and models an internal representation of this information. When the model’s prediction of the forthcoming stimulus is violated, the mismatch signal is generated (Paavilainen et al., 1999; Näätänen et al., 2005; Winkler, 2007).

Importantly, the amplitude of the MMN is modulated by previous experiences and correlates with behavioral discrimination performance. An initial poor differentiation of the deviant and standard stimuli, as well as inaccurate performance, are correlated with a low-amplitude MMN, while active learning to discriminate deviant stimuli results in larger MMN activity (Sams et al., 1985; Novak et al., 1990; Näätänen et al., 1993; Tiitinen et al., 1994; Cheour et al., 2002). Furthermore, learning-dependent changes of the MMN’s amplitude have been demonstrated not only right after discrimination training, but also several days later (Kraus et al., 1995; Tremblay et al., 1998; Menning et al., 2000; Atienza et al., 2002, 2005), a training-dependent long-term effect on pre sensory processing in the auditory cortex. Thus, previous studies have robustly demonstrated that training-induced changes of the MMN amplitude are reliable markers of experience-induced neuroplasticity.

Training-induced enhancement in the MMN is often followed by an increased fronto-central P3a component with a 230–300 ms latency (Draganova et al., 2009). Importantly, P3a, which reflects attentional reorientation to salient, task-irrelevant cues (Escera et al., 1998; Wetzel et al., 2011), is believed to be associated with executive functions (Light et al., 2007; Fjell et al., 2009) and possibly working memory encoding (Bledowski et al., 2004). P3a activity has been linked to both short- and long-term plasticity changes as a result of auditory training (Atienza et al., 2004; Uther et al., 2006; Draganova et al., 2009). Overall, the MMN and P3a components are reliable markers of induced perceptual learning.

We hypothesized that—similar to the effects of classical conditioning—repeated exposure to acoustic incentive cues that predict different monetary outcomes might induce plastic changes in the auditory processing that underlie better discrimination and/or an involuntary attention switch to incentive cues with higher expected values (EVs). Therefore, learning-based neuroplastic changes could be manifested in the increased amplitude of the MMN and/or P3a components. An increased MMN amplitude would indicate a more fine-grained discrimination of the auditory cues, whereas an increased P3a would indicate a stronger reallocation of attention to the cues guided by the prefrontal cortex.

The monetary incentive delay (MID) task is a popular tool for studying the different stages of reward-based learning, from reward anticipation to its delivery (Knutson et al., 2000, 2005). In the traditional version of the MID task, visual stimuli, such as circles, squares, and triangles, are utilized as incentive cues that code the probabilities and magnitudes of outcomes. The MID task allows the manipulation of the EVs, the sum of all possible outcomes of a particular choice multiplied by their probabilities, and reward-prediction errors (RPEs). The modern theory of reinforcement learning (RL) assumes that RPE signals drive the feedback-guided adaptive modification of behavior to environmental change (Sutton and Barto, 1998). In the current study, we investigated the link between neural activity correlated to RPE signals and neural activity correlated to neuroplasticity (MMN and P3a).

To study auditory perceptual learning, we developed an auditory version of the MID task (Krugliakova et al., 2018) where the sounds of different frequencies and intensities were used as incentive cues for signaling the prospective gain’s probabilities and magnitudes. We suggested that a continuous MID task could evoke plastic changes in auditory processing such that processing the incentive cues would be facilitated proportionally to the cues’ EVs. To test this hypothesis, we analyzed feedback-related activity during the MID task. Numerous electroencephalography (EEG) studies have shown that the feedback-related negativity (FRN) component reflects a neural activity that underlies learning and performance monitoring (Holroyd and Coles, 2002; Montague and Berns, 2002; Montague et al., 2004; van Meel et al., 2005; Sambrook and Goslin, 2016). The FRN is a negative deflection with a fronto-central maximum occurring 240–340 ms after receiving negative feedback. According to Holroyd and Coles (2002), the FRN reflects a phasic decrease in dopaminergic activity that disinhibits the anterior cingulate cortex, which signals an RPE (Hajihosseini and Holroyd, 2013). A number of studies have provided evidence for the links between the FRN and mid-frontal theta oscillations with individual behavioral changes (for a review, see Luft, 2014). We recorded the FRN during the MID task and then studied the correlation of the FRN’s amplitude with changes in the MMN and P3a, which was recorded using the oddball paradigm before and after the MID task. Overall, we tested two hypotheses: (I) MID task performance can induce plastic changes in the auditory system as reflected in MMN and P3a amplitude; and (II) individual differences in the plastic changes of the auditory processing can be predicted by the individual differences of the FRN recorded during the MID task. Overall, our findings could clarify a relationship between reward-based learning and sensory plasticity during behavioral adaptations.

## Materials and Methods

### Subjects

Forty-two subjects (17 females) participated in an EEG experiment in which both behavioral and electrophysiological data were collected. Five subjects were excluded from the analysis because of excessive EEG artifacts or too few artifact-free trials (less than 20 trials per trial type; for the same approach, see Marco-Pallares et al., 2011). Data of 37 subjects (15 females, 23 ± 3 years old) were included in the final statistical analysis. All of the subjects were right-handed with normal or corrected-to-normal vision and reported normal hearing; they did not report any history of psychiatric or neurological problems. The experiment was carried out in accordance with the recommendations of the Declaration of Helsinki and its amendments, and the protocol was approved by the ethics committee of the National Research University Higher School of Economics. All subjects gave written informed consent in accordance with the Declaration of Helsinki.

### Study Design

**Figure 1:**
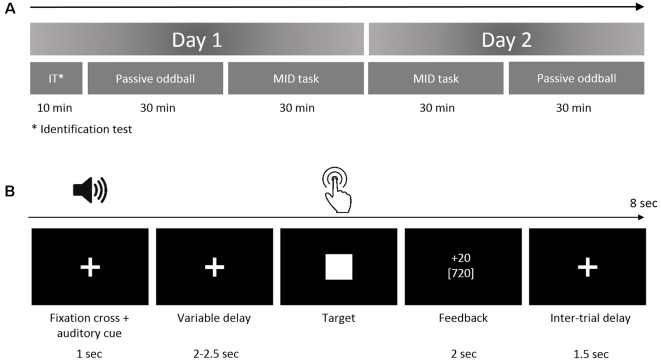
**(A)** Study protocol. The subjects performed the oddball task and monetary incentive delay (MID) task on two successive days. The identification test prior to the first oddball session was designed to ensure that the participants could discriminate among the auditory stimuli. **(B)** The structure of the trial in the auditory version of the MID task. In the beginning of each trial (first box), the participants were exposed to acoustic cues encoding the prospective gain magnitude [4 or 20 Russian rubles (RUB)] and the probability of a win (*p* = 0.80 or *p* = 0.20). After a variable anticipatory delay period (second box, fixation cross, duration = 2,000–2,500 ms), the participants responded with a single button press as quickly as possible after the presentation of a visual target (third box, white target square). Next, 800 ms after the button press (the end of each trial), a feedback screen (fourth box, duration = 2,000 ms) was presented. During the feedback, the top number indicated the amount of money won during that trial, and the bottom number indicated the participant’s total amount won. The overall duration of a single trial was 8 s on average.

The primary goal of the current study was to investigate the effects of a continuous MID task requiring the anticipation and processing of rewards on the neural processing of auditory incentive cues (see Table 1). For this purpose, we designed an experiment consisting of two tasks (the MID task and oddball paradigm) that was presented on two successive days (Figure 1). The rationale to use two MID-task sessions in two subsequent days was that including at least one full night of sleep following the initial acquisition could be beneficial for the individual’s associative leaning (Atienza et al., 2004; Gottselig et al., 2004b; Talamini et al., 2008; Ramadan et al., 2009). The results of both MID-task sessions were included to test which stage of the training would be more crucial for the learning-induced changes in sensory processing: the initial phase of training that can contribute to the overnight memory consolidation or the final phase preceding the retest in the oddball task.

**Table 1:** Acoustic stimuli in the oddball task and monetary incentive delay (MID) task.

Stimuli	Oddball task	MID task	
		Group 1	Group 2
Std	(523 Hz)/70 dB	-	-
Dev_I1F1_	−10/8 semitones (487 Hz)/55 dB	+20 RUB/0.80	+20 RUB/0.20
Dev_I1F2_	+10/8 semitones (562 Hz)/55 dB	+4 RUB/0.80	+20 RUB/0.80
Dev_I2F1_	−10/8 semitones (487 Hz)/80 dB	+20 RUB/0.20	+4 RUB/0.20
Dev_I2F2_	+10/8 semitones (562 Hz)/80 dB	+4 RUB/0.20	+4 RUB/0.80

#### Experimental Procedure

Prior to the experiment, the participants were informed that during each of the two MID task sessions, they had a chance to earn some amount of money and that at the end of the second day, they would receive the largest of the two total gains.

##### Day 1

At the beginning of each experiment, the ability of participants to discriminate between four auditory stimuli was tested during an identification test. Next, the participants performed the first session of a passive oddball task where the four above-mentioned auditory stimuli were used as deviant stimuli.

At the beginning of the MID task, the participants were instructed about the meaning of each auditory stimuli as an “acoustic cue” that coded a specific EV. Finally, the participants performed the first session of the MID task where four auditory stimuli were used as incentive cues with different EVs.

##### Day 2

At approximately the same time of the day, the participants performed the MID task and the oddball task for the second time. Both the MID and the oddball tasks were analogous across the two experimental days. At the end of the second day, the subjects were informed of the monetary gain on the first and the second day and were given the exact amount of the largest gain. The duration of the studies, including the preparation time, was 2 h on the first experimental day and 1.5 h on the second day (Figure 1A).

##### Identification Test

The identification test was designed to ensure that the participants were sufficiently good at discriminating among auditory stimuli that were later used as incentive cues during the MID task. As shown in the previous studies of auditory discriminative training, the training-evoked changes in the MMN could be observed only if subjects could initially discriminate among the various tones relatively well (Gottselig et al., 2004a). The participants were instructed to press a button corresponding to the delivered sound. The sound descriptions and target buttons were displayed on the screen during the task. The participants received positive and negative visual feedback to facilitate learning. The EEG session started when a subject successfully identified 8 out of 10 consecutive sounds. On average, the participants made more mistakes in frequency identification (4.08 ± 0.80; the mean ± the standard error of the mean) than in intensity identification (1.78 ± 0.36) and in simultaneous frequency and intensity identification (1.35 ± 0.39).

##### Auditory MID Task

During the auditory MID task (Figure 1B), the participants were exposed to acoustic cues that encoded the prospective gain magnitude [4 or 20 Russian rubles (RUB) ≈ 0.06 or 0.20 USD] and the probability of a win (*p* = 0.80 or *p* = 0.20). After a variable anticipatory delay period (2,000–2,500 ms), the participants responded with a single button press immediately after the presentation of a visual target (white square, see Figure 1B). Next, the feedback (duration = 2,000 ms, delay = 800 ms) notified the participants whether they had won or missed money during that trial and showed their cumulative total. The 800-ms delay before the feedback was aimed at eliminating the effects of the visual target on feedback-locked ERPs. The overall duration of a single trial was ~8 s. The probability of a win was manipulated by altering the average target duration through an adaptive timing algorithm that followed the subjects’ performance such that they would succeed in ~80% of the high-probability trials and in ~20% of the low-probability trials (Knutson et al., 2005). Positive outcomes occurred in an average of 58 ± 6 trials out of 76 high-probability trials and an average of 14 ± 3 trials out of 76 low-probability trials.

To encode prospective reward probability and magnitude, the auditory cues had two levels of frequency and two levels of intensity. The probability and magnitude of the reward were encoded differently in two experimental groups. In Group 1 (*n* = 19), the intensity of the acoustic cue encoded the gain’s magnitude, while the frequency encoded the gain probability. In Group 2 (*n* = 18), the encoding of the gain magnitude and gain probability was reversed. To eliminate the effects of the stimuli’s physical parameters on the ERPs, we polled the data of the two experimental groups.

### Auditory Stimuli and Oddball Paradigm

To probe the learning-related neuroplasticity of the auditory processing, the subjects participated in two identical passive oddball tasks, with the first session of the oddball task performed on Day 1 before the first MID session, while the second session of the oddball task was performed after the second MID session on Day 2 (Figure 1A). The standard stimuli in the oddball paradigm were composed of three sinusoidal partials (523, 1,046, and 1,569 Hz, with a fundamental frequency corresponding to C5 of the Western musical scale, intensity = 70 dB). Four distinct deviant tones (Table 1) differed from the standard tone in both frequency and intensity such that the probability of an increment or decrement was even. The deviants differed from the standards in their frequency by +10/8 and −10/8 semitones on the Western musical scale (fundamental frequencies 562 Hz for the higher and 487 Hz for lower deviant tones). The intensity of the deviants was either smaller or larger than the standard (70 dB) by 15 dB and 10 dB, respectively (55 dB and 80 dB). All stimuli lasted 200 ms (including 5 ms rising and falling times). The stimuli were generated with Praat acoustics software (Boersma, 2001).

Importantly, the same four deviant oddball stimuli were also used as acoustic reward-predictive cues for the auditory MID task. The acoustic cues signaled high or low prospective reward probabilities (0.80 and 0.20, correspondingly) and high or low prospective reward magnitudes (4 or 20 RUB, correspondingly), as illustrated in Figure 1B. For example, in Group 1, the deviant stimulus Dev_I1F1_ (487 Hz/55dB) signaled an opportunity to receive 20 RUB with a 0.80 probability (see the details of the reward-predictive cues in Table 1).

During the oddball tasks, infrequent deviant stimuli were pseudo-randomly interspersed with a standard stimulus presented with a probability (*P*_std_) of 0.80 and with an 800 ± 100 ms onset asynchrony. Each deviant type (Dev_I1F1_, Dev_I1F2_, Dev_I2F1_, and Dev_I2F2_) was presented as every fourth, fifth, or sixth tone with the same probability (*P*_dev_ = 0.20/*4* = 0.05). Two successive deviants were always of a different type (an example of the progression of tones during a session is as follows: Dev_I2F2_ – Std – Std – Std – Dev_I1F1_ – Std – Std – Std – Std – Std – Dev_I2F1_ – Std – Std – Std – Std – Dev_I1f1_ – Std –…). Overall, each oddball session consisted of 2,400 tones (session duration = 30 min), and each of the four deviant stimuli was presented 120 times. Each session started with a training session of four standard stimuli. During passive oddball sessions, the subjects read a book of their own choice.

### EEG Data Acquisition

EEG data were recorded with 28 active electrodes (Brain Products GmbH, Gilching, Germany) according to the extended version of the 10-20 system: Fp1, Fp2, F3, F4, C3, C4, P3, P4, O1, O2, F7, F8, T7, T8, P7, P8, Fz, Cz, Pz, Oz, FC1, FC2, CP1, CP2, FC5, FC6, CP5, and CP6. The active channels were referenced against the mean of two mastoid electrodes to display the maximal MMN and FRN response at the frontal electrode sites. The electro-oculogram was recorded with electrodes placed on the outer canthi and below the right eye. Data were acquired with a BrainVision actiCHamp amplifier (Brain Products GmbH, Gilching, Germany) and sampled at 500 Hz. Impedance was confirmed to be less than 5 kΩ in all electrodes prior to recording.

### EEG Data Analysis

EEG signals were preprocessed with BrainVision Analyzer 2.1 (Brain Products GmbH, Gilching, Germany). The EEG was filtered offline (passband 1–30 Hz, notch filter 50 Hz), and then, an independent component analysis (ICA)-based ocular artifact correction was performed. After a manual inspection of the raw data for the remaining artifacts, the data were segmented into epochs of 600 ms and a 100-ms prestimulus epoch. Each trial was baseline corrected to an average activity between −100 and 0 ms before stimulus onset. Epochs, including voltage changes exceeding 75 μV at any channel, were omitted from the averaging. For the oddball task and MID task, the epochs were averaged for two sessions separately. The time windows chosen for the statistical analysis of the ERP components were based on a visual inspection of the grand-average waveforms and previous studies. The ERP components were defined either as the local maximum (P3a) or local minimum (MMN and FRN) of the difference waveform. Once a peak was identified, the amplitude over a ±10-ms window around this peak was averaged individually and then averaged across the participants. We complemented this analysis with the measurement of the area under the ERP curve (AUC, μV *ms), which provides a more precise measure of the overall magnitude of the brain’s response (Kappenman and Luck, 2012) in cases of the multipeak nature of the ERP components. The AUC was computed as the approximate integral using the Matlab function *trapz.m*.

All statistical analyses were performed using Matlab 2015a and SPSS software package (22.0).

#### Analysis of the MMN and P3a Components Recorded During the Oddball Task

To study experience-induced plastic changes, we analyzed the MMN and P3a components before and after the MID task training. The data were segmented for five types of trials: standard stimulus and four types of deviants (Dev_I1F1_, Dev_I1F2_, Dev_I2F1_, and Dev_I2F2_). The difference waveforms were derived by subtracting the averaged response to the standard stimulus from the averaged response to each type of deviant stimulus. The MMN peak amplitude was identified as the most negative peak in the difference response occurring at 80–250 ms poststimulus onset at the Fz electrode (Näätänen et al., 2007). The P3a peak amplitude was identified as the most positive peak of the difference curve occurring at 180–300 ms poststimulus onset at the same electrode (Seppänen et al., 2012).

For the oddball task, three-factor repeated measures analyses of variance (ANOVAs) with *session* (session 1 vs. session 2), *probability* (low probability vs. high probability), and *magnitude* (small magnitude vs. big magnitude) as the within-subject variables were conducted separately for the MMN and P3a amplitudes. We used the Greenhouse–Geisser correction to estimate the *p* values. The level of significance was set to *p* < 0.05.

#### Interaction of FRN With MNN and P3a

**Figure 2:**
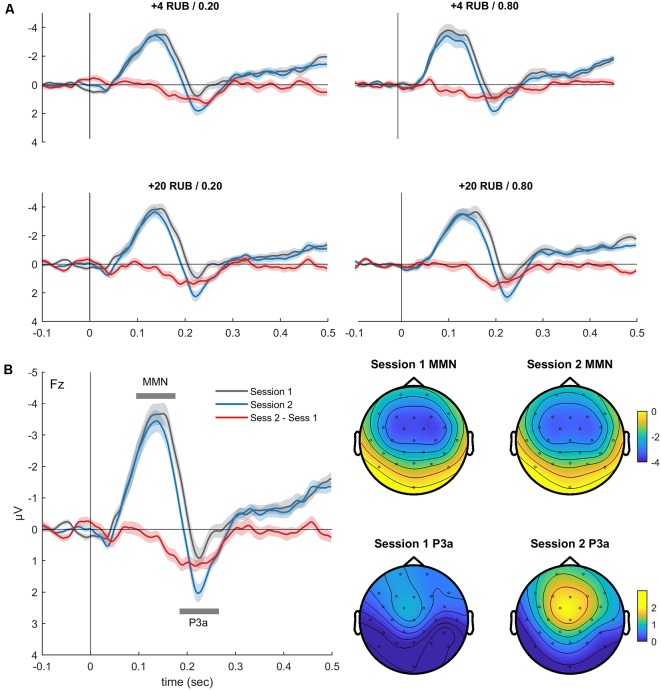
**(A)** Grand-averaged difference waveforms (Fz, deviant minus standard) superimposed for the two oddball sessions before and after the MID task. The event-related potentials (ERPs) are presented for all four deviants, which also signaled combinations of the magnitude and probability of gain in the MID task. **(B)** Difference waveforms (Fz, left) derived by averaging the ERPs across four conditions, and corresponding scalp topography (right) of the mismatch negativity (MMN) and P3a during oddball sessions 1 and 2. Shaded area around curves represents standard error of the mean (SEM). The topographic maps indicate the voltage distribution of the mean amplitude in the 110–130-ms (MMN) and 220–240-ms (P3a) time windows.

We analyzed whether the changes in the MMN and the P3a (between session 1 and session 2) induced by the MID task varied as a function of the FRN amplitude registered during the MID task session 1 and session 2 (FRN1 and FRN2). First, to calculate the *difference MMN*, or dMMN, and the* difference P3a*, or dP3a, we subtracted the difference waveforms in session 1 from the difference waveforms in session 2 (red lines in Figure 2A) and calculated the average amplitudes of the dMMN (Fz, 50–200-ms time window) and dP3a (Fz, 150–280-ms time window). Second, we calculated a standard FRN separately for both the MID task sessions by subtracting the ERPs of all the positive outcomes from the ERPs of all the negative outcomes (omission of gain). The amplitudes of the FRN (Cz) were quantified within a 230–350-ms time window poststimulus onset. Finally, to measure the relationship between the dMMN and dP3a (oddball task) and the FRN (MID task session 1 and session 2), we calculated the Spearman correlations between these two classes of variables. We used Cook’s distance to identify any outliers. Cases with Cook’s distances bigger than 4/*n* were excluded from further analysis (Bollen and Jackman, 1985).

## Results

### Training-Induced Neuroplasticity: Comparison of ERPs in Oddball Sessions 1 and 2

Figure 2A shows the auditory difference waveforms that were calculated separately for sessions 1 and 2 of the oddball task. The difference waveforms were calculated by subtracting the ERPs of each deviant (Dev_I1F1_, Dev_I1F2_, Dev_I2F1_, or Dev_I2F2_) from the ERPs of the standard stimuli. Importantly, these four deviants also signaled different rewards during the MID task performed between the two sessions of the oddball task. A negative deflection (MMN) peaking around 120 ms after stimulus onset and a positive deflection (P3a) peaking around 230 ms are distinctly observed in all fronto-central difference waveforms. The latencies and fronto-central distribution clearly indicate the neural generators of the MMN and P3a.

The three-way ANOVA yielded a main effect of the *session* variable for the MMN amplitude (*F*_(1,36)_ = 5.80, *p* = 0.02, $\eta_{\text{p}}^{2}$ = 0.14), indicating a slight reduction of the MMN in session 2 (−3.98 ± 0.34 μV) compared with session 1 (−4.40 ± 0.34 μV). The main effect of the *session* variable was also significant for the P3a amplitude (*F*_(1,36)_ = 29.23, *p* < 0.001, $\eta_{\text{p}}^{2}$ = 0.45), reflecting increased P3a in session 2 (2.16 ± 0.24 μV) compared with session 1 (1.31 ± 0.25 μV). The main effects of the *magnitude* and *probability* variables were not significant for both the MMN and P3a amplitudes. No significant interactions between the factors were observed. Thus, only the P3a component showed an increased amplitude in session 2 compared with session 1, which may indicate learning-related plastic changes in the auditory system.

Figure 2A illustrates the similarity of the difference waves’ learning-related changes for all deviants. Therefore, for further analysis, we pooled together the difference waveforms obtained for the four types of deviants (Figure 2B). Interestingly, we observed a significant reduction of the MMN AUC on the second day (*t*_(35)_ = 2.87, *p* = 0.007), reflecting shorter latency and a smaller duration of the MMN.

To investigate whether such a decrease can be explained by the presentation of repetitive stimulus, we analyzed the changes of the ERPs to oddball standards across two sessions. Notably, we found no changes in the amplitude of the ERPs to standard sound on the second day (Supplementary Figure S1). In addition, an analysis of the ERPs to the oddball deviants (without subtracting the standards) demonstrated that on the second day, there was a clear decrease of N200 amplitude that was associated with an enlargement of P300 (all *p* < 0.05). This effect might partially explain the “shortening” of MMN on the second day (Supplementary Figure S1). Overall, the MID task, rather than just exposure to sounds during the oddball task, induced changes of the ERPs to auditory monetary cues.

### Relationship of RL Signals and Neuroplasticity: Correlation Analysis

**Figure 3:**
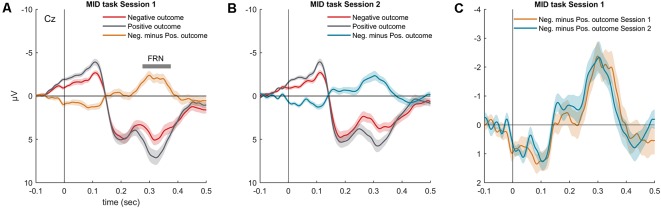
Grand-averaged visual ERP waveforms (Cz) superimposed for the outcomes with different valences (negative outcome, positive outcome) and the difference waveform recorded during the MID task **(A)** session 1 and **(B)** session 2. **(C)** Superimposed difference waveform (Cz, negative minus positive outcomes) for two sessions of the MID task. Shaded area around curves represents SEM.

As expected, omission of a gain during the MID task evoked the FRN component (Figures 3A,B). In both MID task sessions, the FRN appeared as a negative difference wave, with a maximum between 200 and 400 ms following the feedback onset. We did not observe any difference in the FRN amplitude across the two sessions of the MID task: *t*_(36)_ = 0.01, *p* = 0.99 (Figure 3C). Furthermore, we found no significant difference in the FRN, AUC across the two sessions: *t*_(36)_ = 0.35, *p* = 0.72. The detailed analysis of the effect of probability, magnitude and valence of the outcome on the FRN amplitude can be found in Krugliakova et al. (2018). To sum it up, the FRN was modulated by all three factors. Although the effect of the probability was significant only for the gain trials, the effect of the magnitude was significant for both the gain and omission of the gain trials.

**Figure 4:**
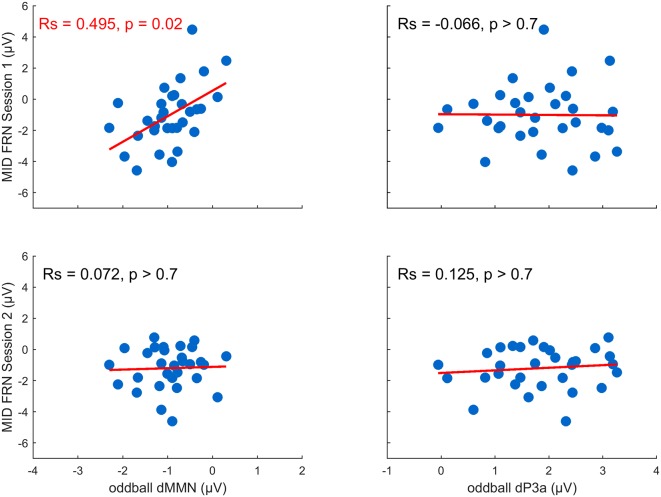
Training-related changes in the MMN and P3a amplitude as a function of the feedback-related negativity (FRN) recorded in the first and the second MID task sessions (the *p*-values were FDR-corrected).

We tested our hypothesis that individual differences in auditory plasticity reflected in the dMMN and dP3a amplitudes can be predicted by individual differences in the FRN recorded during the MID task sessions (FRN1 and FRN2; Figure 4). The correlation analysis yielded a significant relationship between the dMMN and FRN1 (*R*_S_ = 0.50, *p* = 0.02, FDR corrected), indicating that a larger dMMN was associated with a larger FRN during the MID tasks on the first day. Neither the dP3a and FRN nor the dMMN and FRN2 amplitudes were significantly correlated (*p* > 0.70, FDR corrected). Overall, our results show that plastic changes in the auditory processing correlated with the RL signals recorded during the first MID task training session but not during the second one.

In addition, we tested if the individual differences of the dMMN could be explained by the degree of a subject’s involvement in the MID task. To do this, we performed an exploratory analysis of the reaction times (RTs) in trials with different outcome probabilities. Because the probability of a gain was manipulated by altering the average target duration by an adaptive timing algorithm, we suggested that the subjects who paid attention to the incentive cues would react to the white target square faster in low-probability trials when compared with the high-probability trials. Thus, the subjects’ involvement in the MID task could be indexed by the difference in the RTs in trials with different outcome probabilities. Therefore, we calculated the dRT: the RTs in trials with low-probability outcomes *minus* the RTs in trials with high-probability outcomes, normalized by the average RTs (a more detailed analysis of the behavioral data of this dataset was published in Krugliakova et al., 2018). We quantified the correlation among the dMMN, P3a, and dRT and observed a positive correlation between the dMMN and dRT (*R*_S_ = 0.39, *p* = 0.02) but none between the dP3a and dRT (*R*_S_ = −0.07, *p* > 0.7; Supplementary Figure S2). Thus, subjects who showed a larger increase of the MMN amplitude in session 2 relative to session 1 showed a larger difference in their RTs in the trials with different outcomes probabilities. Therefore, the individual differences of the dMMN could be partially explained by the attention that the subjects paid to the incentive cues during the MID task.

## Discussion

In the current study, we investigated whether intensive training in the MID task-induced plasticity in sensory processing. As a result, we identified a significant training-induced increase of the P3a component associated with the processing of incentive cues but not of the MMN component. A more detailed analysis of the individual differences demonstrated a large variability in training-induced changes to the MMN. Interestingly, we found a significant correlation between the individual differences in the training-induced changes to the MMN and the individual differences in the FRN, which is a neural marker of the RL elicited during the MID task.

Contrary to our prior hypothesis regarding the increase of the MMN amplitude as a function of training-induced plasticity, in the present study, the MMN decreased on average after two sessions of the MID task. Many studies have shown an increase in the MMN during training (e.g., Atienza et al., 2004; Gottselig et al., 2004a, b). However, a few recent studies have shown that the MMN amplitude could decrease after training (Müller et al., 2002; Perez et al., 2017). In other studies where significant reductions in sensory ERPs after training have been observed (Berry et al., 2010; Miyakoshi et al., 2012), the authors of these articles explained the MMN decrease as being a result of neural adaptation. In addition, a few previous experiments demonstrated an attentional modulation of the MMN (Woldorff et al., 1991; Sussman et al., 2014; Auksztulewicz and Friston, 2015) and the importance of stimulus significance in sensory processing (Bradley, 2009). In light of this, we hypothesized that the direction of the MMN changes could be associated with a different level of the subjects’ motivation to distinguish among the different auditory cues during the MID task. Indeed, individual differences of the dMMN in the oddball task could partially account for the individual differences in the RTs during the performance of the MID task across the trials that had different outcome probabilities. In the MID task, the subjects who showed a larger MMN in session 2 than in session 1 of the oddball task responded faster in the trials with a low outcome probability than in the trials with a high outcome probability. Therefore, we speculate that an MMN decrease in session 2 compared with in session 1 might be associated with the decreased attention of the subjects to the auditory cues during MID task performance, which could result in habituation and neural adaptation to auditory stimuli. It should be noted, however, that the observed decrease in the MMN amplitude could be at least partially driven by changes in a stimulus-specific N100 response in addition to changes in the “pure” MMN component. In future studies, this problem can be tackled by using a roving oddball paradigm, where there are no acoustic differences between the standard and deviant.

Nevertheless, we found a link between training-induced changes of the MMN and FRN recorded during the first session of the MID task. Previous studies have demonstrated that the size of the FRN predicted the effectiveness of the learning (for a review, see Luft, 2014). Most of these studies used paradigms that required learning probabilistic associations rather than error-based learning (Yasuda et al., 2004; Frank et al., 2005; Cohen and Ranganath, 2007; Philiastides et al., 2010; van der Helden et al., 2010; Arbel et al., 2013; for a review, see Luft, 2014). In the current study, the FRN recorded during the first session of the MID task correlated with the training-induced changes of the MMN evoked by incentive cues during the oddball task. This indicates that the participants who demonstrated a larger FRN during the first session of the MID task may also have demonstrated an increased MMN, while the participants with smaller FRNs would show a decreased MMN. The training-induced increase of the MMN in subjects with a pronounced FRN might indicate a selectively induced plasticity of the auditory cortex driven by performance in the MID task. Interestingly, the FRN recorded during the first MID session predicted subsequent changes in the MMN amplitude better than the FRN recorded immediately prior to the second oddball session. One possible explanation for this is that the first MID task session was followed by a sufficient amount of time for effective top-down modulation of auditory processing, which, as has been shown, also benefits from sleep (Atienza and Cantero, 2001; Atienza et al., 2004).

Contrary to our expectations, the decrease in the MMN amplitude was not specific to low EVs. This insensitivity of the MMN’s changes to manipulations of the EVs could be explained by limitations of the standard MID task. According to the auditory version of the MID task, all cues should be equally important for optimal performance in the task. In other words, to react specifically to one of the incentive cues, one would need to discriminate this cue from all other incentive cues. Thus, the participants should learn all of the cues equally well, regardless of their EVs. This interpretation would be in accordance with the predictions of the reverse hierarchy theory of perceptual learning (Ahissar et al., 2009): for perceptual learning to occur, specific learning paradigms that are optimal for the modification of sensory representations and that result in more accurate perceptions need to be utilized. Unfortunately, the standard MID task is neither designed to test the effects of perceptual learning as training-induced improvements in discrimination nor the controls for sensitization as a nonspecific facilitation of stimuli identification. To further study the effects of reward-based learning on sensory plasticity, a modification of the MID task would be necessary, for example, better discrimination of incentive cues with higher EVs being relevant for task performance.

Learning-related changes to the MMN are frequently accompanied by changes to the P3a. The MMN has been linked to the perceptual processes underlying stimulus discrimination, and it is often used as an index of central auditory system plasticity (for a review, see Näätänen et al., 2007), whereas the P3a manifests the allocation of involuntary attention to the relevant stimuli (Polley, 2006). For example, learning foreign-language phonemes evokes correlated MMN-P3a changes (Shestakova et al., 2003). In a recent review, Jääskeläinen et al. (2011) proposed a model in which rapid plasticity can support not only sensory and short-term memory, but also selective and involuntary attention and perceptual learning, depending on the input type (bottom-up vs. top-down). Recent results of human experimental studies fit this model well. For example, Seppänen et al. (2012, 2013) compared the short-term plasticity effect on involuntary attention using the P3a component of ERPs between musicians and nonmusicians. During passive exposure to sounds after an active discrimination session, the musicians showed a habituation of the P3a, while nonmusicians showed an enhancement of the P3a between blocks. Corroborating this finding, a number of studies showed congruent dynamic changes in the MMN and P3a, suggesting that the MMN-P3a complex is an index of involuntary attention control (Friedman et al., 1998; Debener et al., 2005; Barry et al., 2016). Interestingly, in the current study, the degree of the amplitude changes of the P3a and MMN were not in line with these previous studies. Unlike the MMN, we observed an increase in the P3a amplitude in the second oddball session across all four types of incentive cues. The P3a result without an MMN effect could be explained by a top-down process that mediated experience-induced plasticity, which, in turn, resulted in the enhanced change detection during the oddball task, as was confirmed in the study by Seppänen et al. (2012).

## Study Limitations

There are a number of limitations to the current study that should be noted. First, using the standard MID task, we were not able to address how associative learning affects the sensory processing of stimuli with different incentive values. A single-block design does not allow for assigning different relevance levels to incentive cues, making them equally important for the correct task performance. One way to tackle this problem would be to use a multiple block design where the discrimination of different sounds would be task relevant only for some of the blocks. Second, we did not include a second identification task after the second MID task because we observed sufficient discrimination prior to the first MID task. Thus, in our paradigm, we cannot expect a significant change in the number of mistakes because of the ceiling effect in the accuracy. However, a lack of the measure of the performance changes tempers our interpretation of the link between the EEG markers and potential behavioral benefits. Third, to optimize the auditory ERP data collection during the oddball task, it would be beneficial to use a multi-feature oddball paradigm, such as Optimum-1, allowing an efficient recording of brain responses to several acoustic feature changes within a very short recording time (Näätänen et al., 2004). Finally, 28-channel EEG does not provide a sufficient space resolution for the source reconstruction. Thus, in the current study, the conclusions regarding the localization of the observed effects and involvement of particular brain networks should be regarded with caution.

## Conclusion

In the current study, we tested whether repeated exposure to the stimuli that signal different incentive values in the MID task changes their sensory processing when tackled in the oddball tasks. In the absence of the group MMN effect, we observed learning-related changes of the P3a, indicating a stronger reallocation of attention to the incentive cues. The correlational analysis of individual MMN amplitudes with the MID-session FRN responses revealed that a stronger RL signal was associated with a more fine-grained discrimination of the incentive cues.

Overall, our results showed that plastic changes associated with better discrimination could be sensitive to the continuing valuation of incentive cues that leads to enhanced involuntary attention switching. Further studies will be needed to investigate whether auditory sensory processing may depend on the history of previous decisions.

## Ethics Statement

The experiment was carried out in accordance with the recommendations of Declaration of Helsinki and its amendments, and the protocol was approved by the ethics committee of the National Research University Higher School of Economics. All subjects gave written informed consent in accordance with the Declaration of Helsinki.

## Author Contributions

AS, VK, YS and EK designed the experiment. EK, AG and TF recruited subjects, collected and pre-processed the data. EK, AG, TF, VM and AS performed the analysis of the data. EK, AG, YS, VK, VM and AS wrote the article. All authors approved the final version.

## Conflict of Interest

The authors declare that the research was conducted in the absence of any commercial or financial relationships that could be construed as a potential conflict of interest.

## Supplementary Material

FIGURE S1 Grand-averaged auditory ERP waveforms (Fz) for the standard sound and four types of deviants (without standard subtraction) superimposed for two oddball sessions before and after the MID task.

FIGURE S2 Training-related changes in the MMN and P3a amplitudes as a function of the dRT (difference in reaction time: the RT in low-probability minus the RT in high-probability trials, normalized by the average RT).
