# Supplementary figures and images for: The Monetary Incentive Delay (MID) Task Induces Changes in Sensory Processing: ERP Evidence
Source: Front Hum Neurosci. 2019 Nov 1;13:382. doi: 10.3389/fnhum.2019.00382 (PMC6839045; doi:10.3389/fnhum.2019.00382)

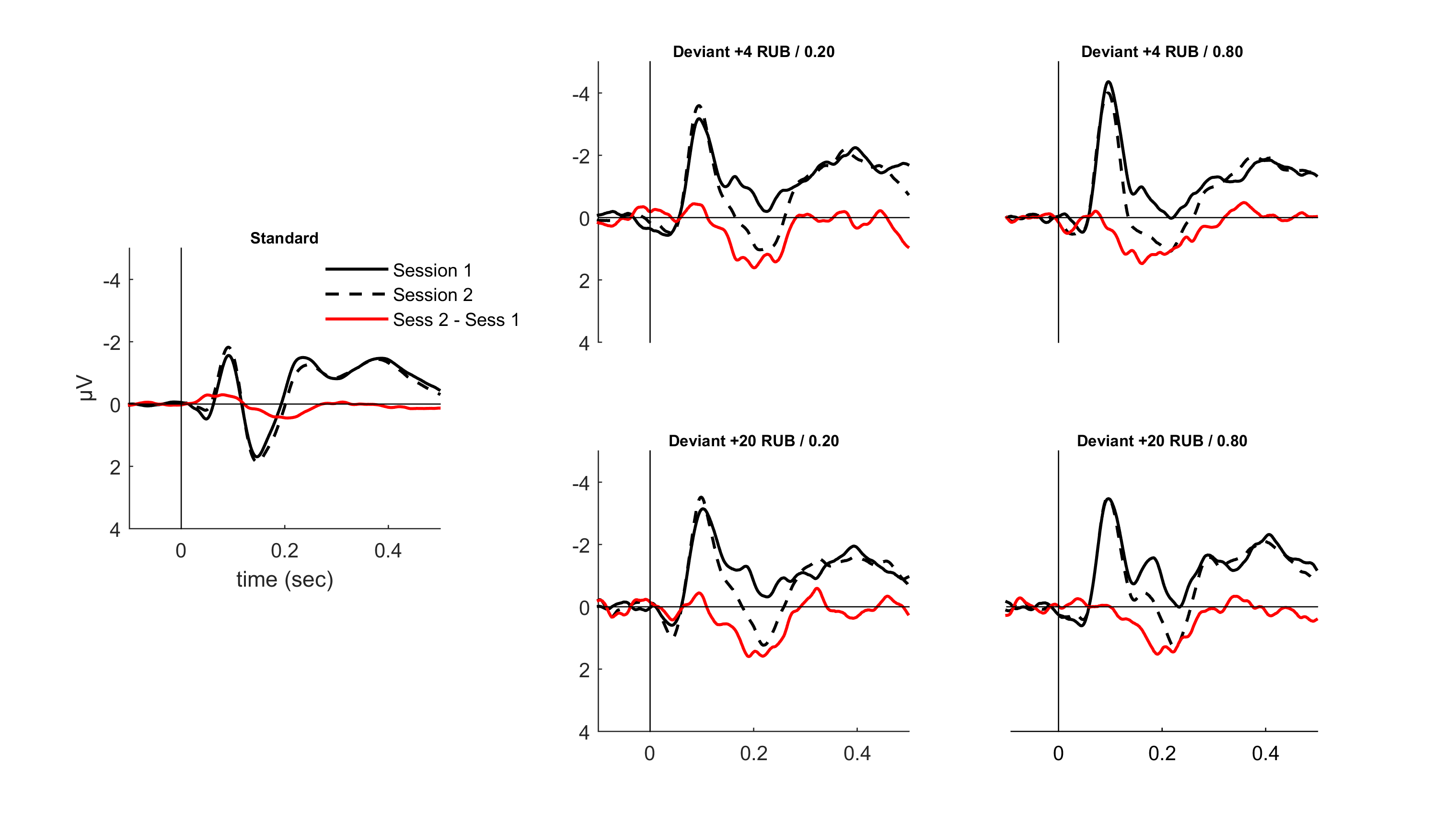

Supplement: FIGURE S1 — Grand-averaged auditory ERP waveforms (Fz) for the standard sound and four types of deviants (without standard subtraction) superimposed for two oddball sessions before and after the MID task. [file Image_1.TIF]

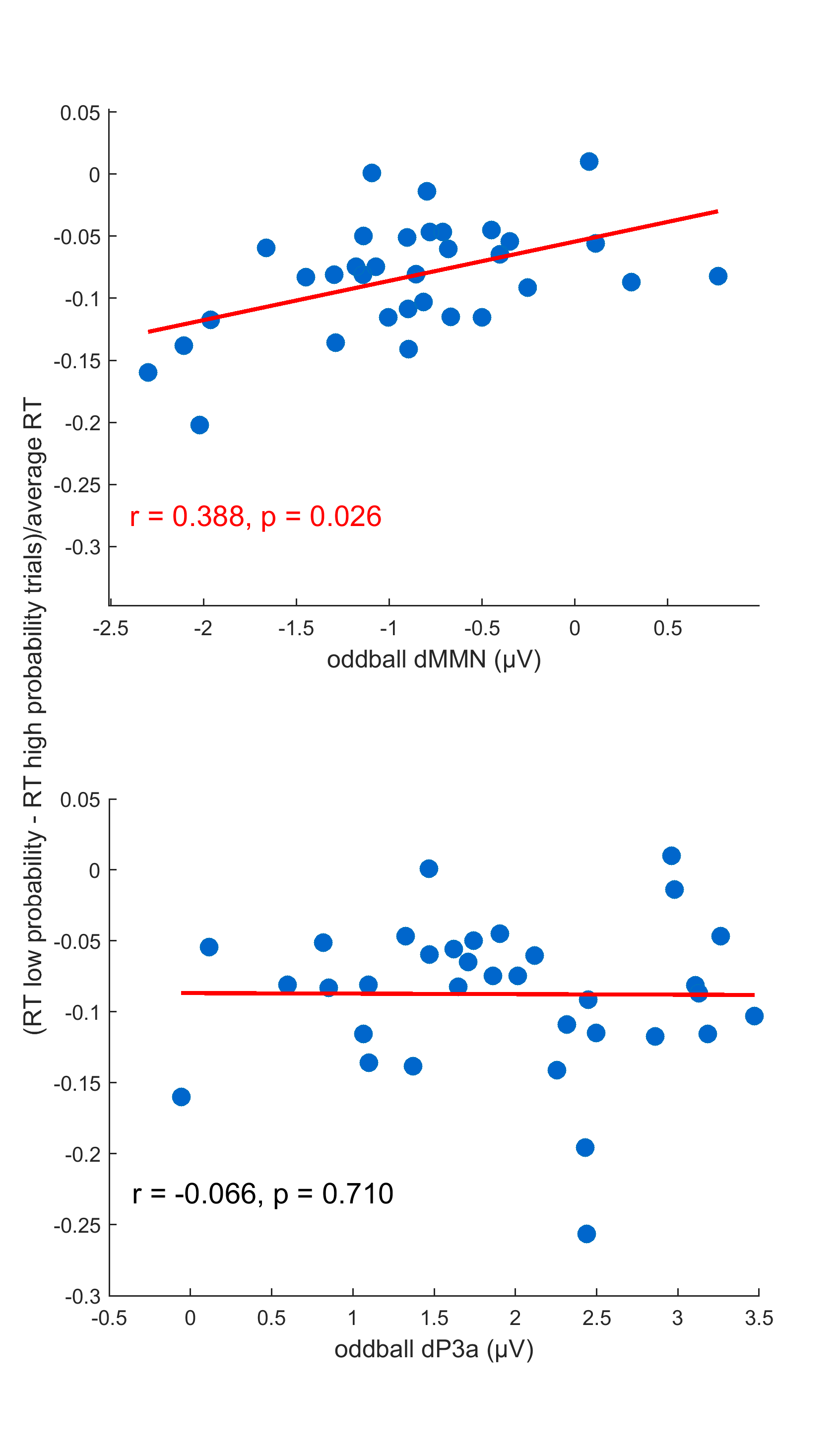

Supplement: FIGURE S2 — Training-related changes in the MMN and P3a amplitudes as a function of the dRT (difference in reaction time: the RT in low-probability minus the RT in high-probability trials, normalized by the average RT). [file Image_2.TIF]
